# Supplementary material for: Development and validation of deep learning algorithms for scoliosis screening using back images
Source: Commun Biol. 2019 Oct 25;2:390. doi: 10.1038/s42003-019-0635-8 (PMC6814825; doi:10.1038/s42003-019-0635-8)
Supplement: Supplementary file 1 — Supplementary Information [file 42003_2019_635_MOESM1_ESM.docx]

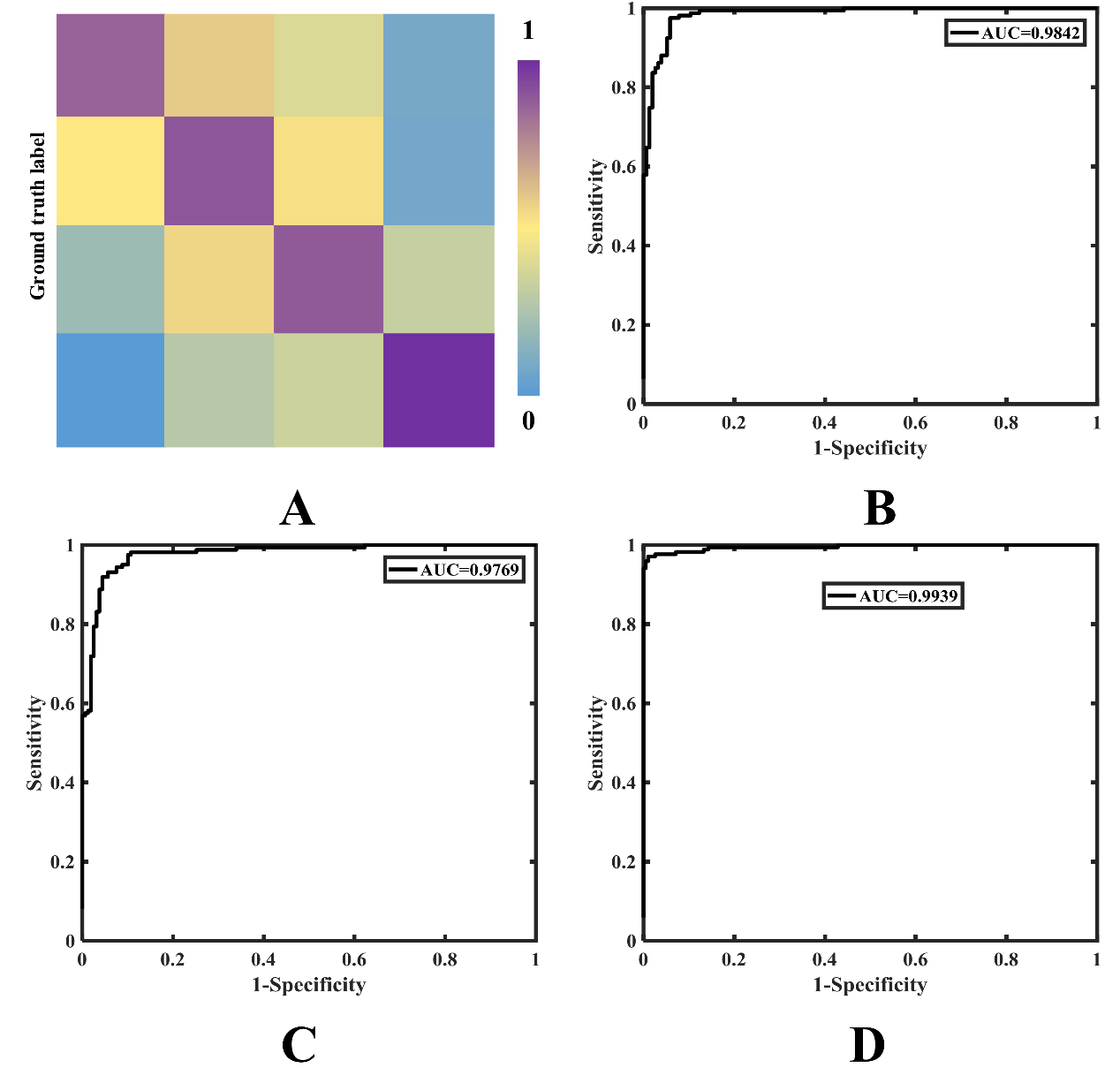


**Supplementary Figure 1. Results of the four additional experiments. A**, Confusion matrix of the four classes of classifications (0 - 9°, 10 - 24°, 25 - 44°, ≥ 45°); **B**, ROC curve of the binary classification (20 - 44°, ≥ 45°); **C**, ROC Curve of the Binary Classification (25 - 44°, ≥ 45°); **D**, ROC curve of the binary classification (0 - 44°, ≥ 45°).


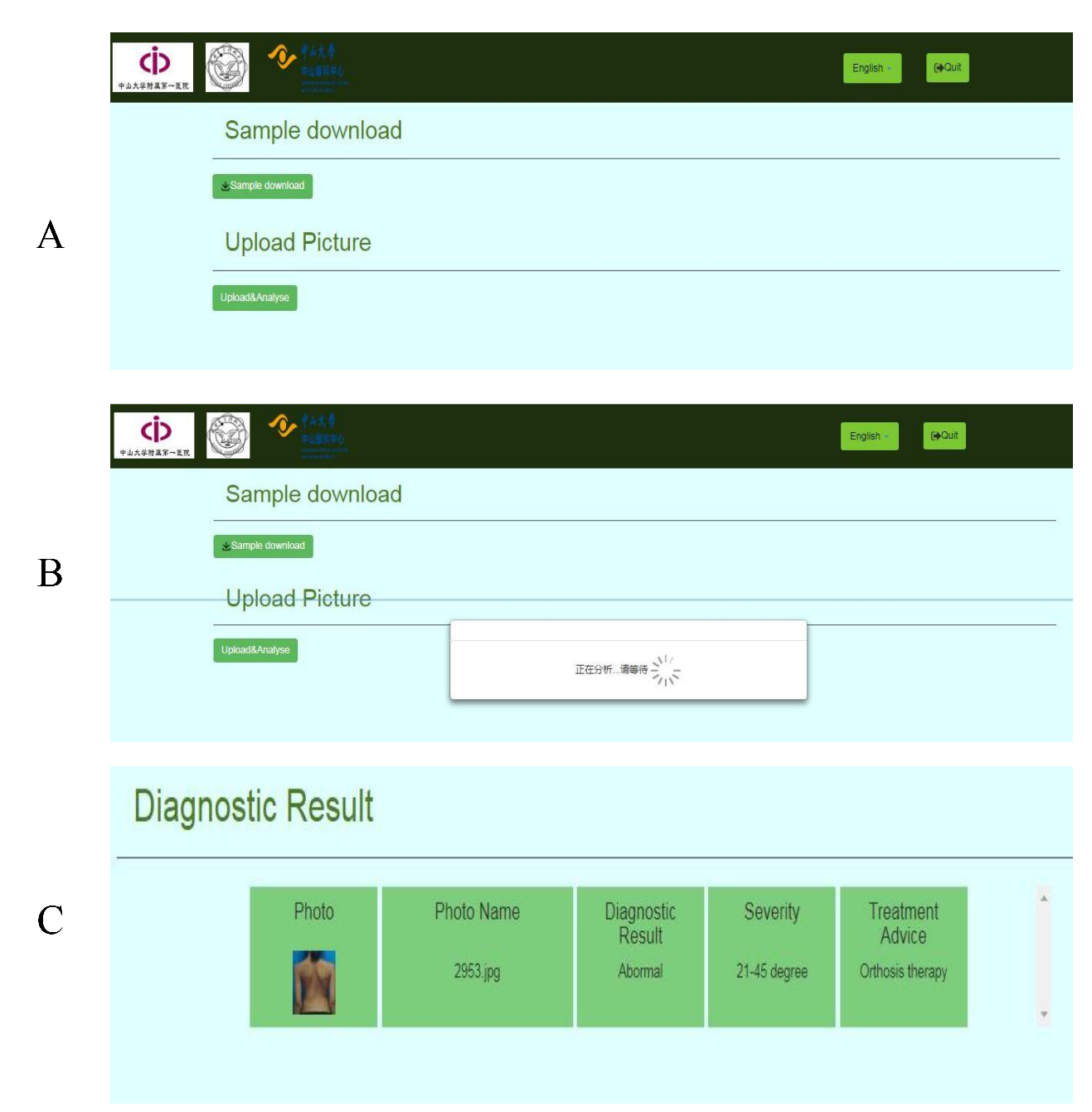


**Supplementary Figure 2. Website for self-screening of scoliosis**. **A**, Website appearance. **B**, Photograph analysis conditions. **C**, Output diagnostic results from the artificial intelligence website.


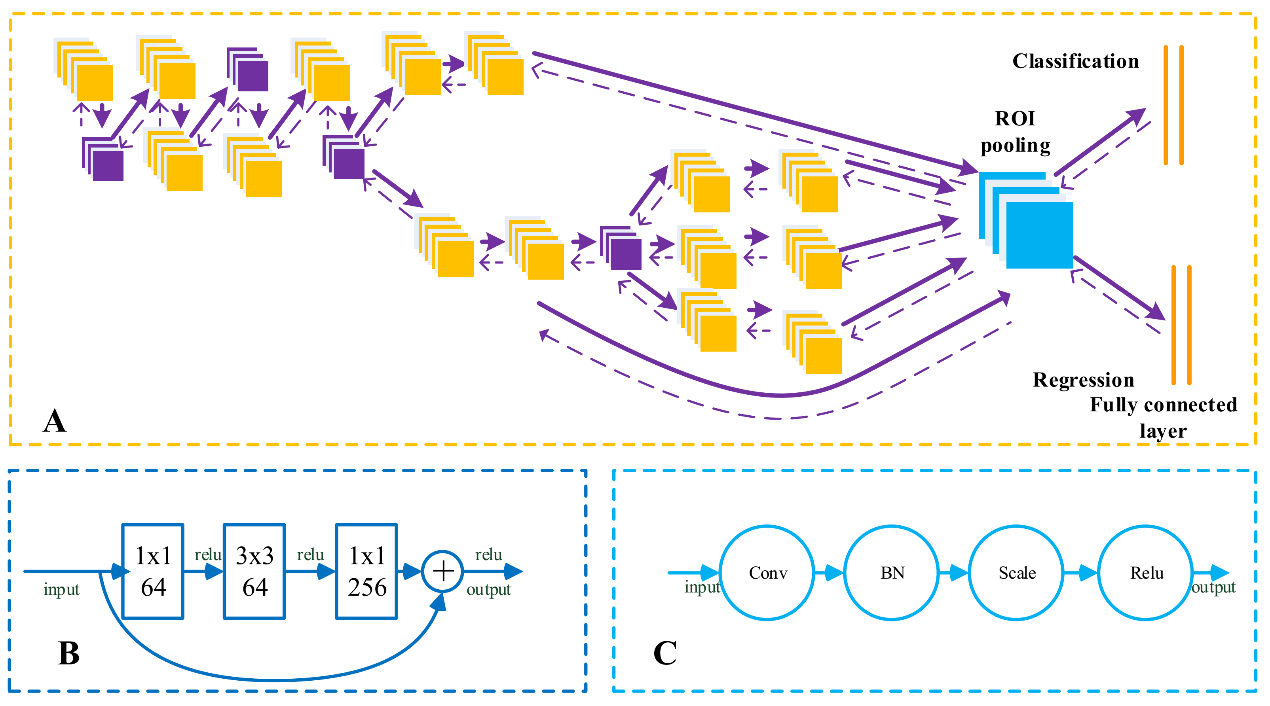


**Supplementary Figure 3. The architecture of Faster-RCNN, the residual block and the batch normalization block of Resnet. A**, Architecture of Faster-RCNN, where yellow squares and purple squares are convolutional kernels and pooling operations; **B**, Residual block; **C**, Batch normalization block.


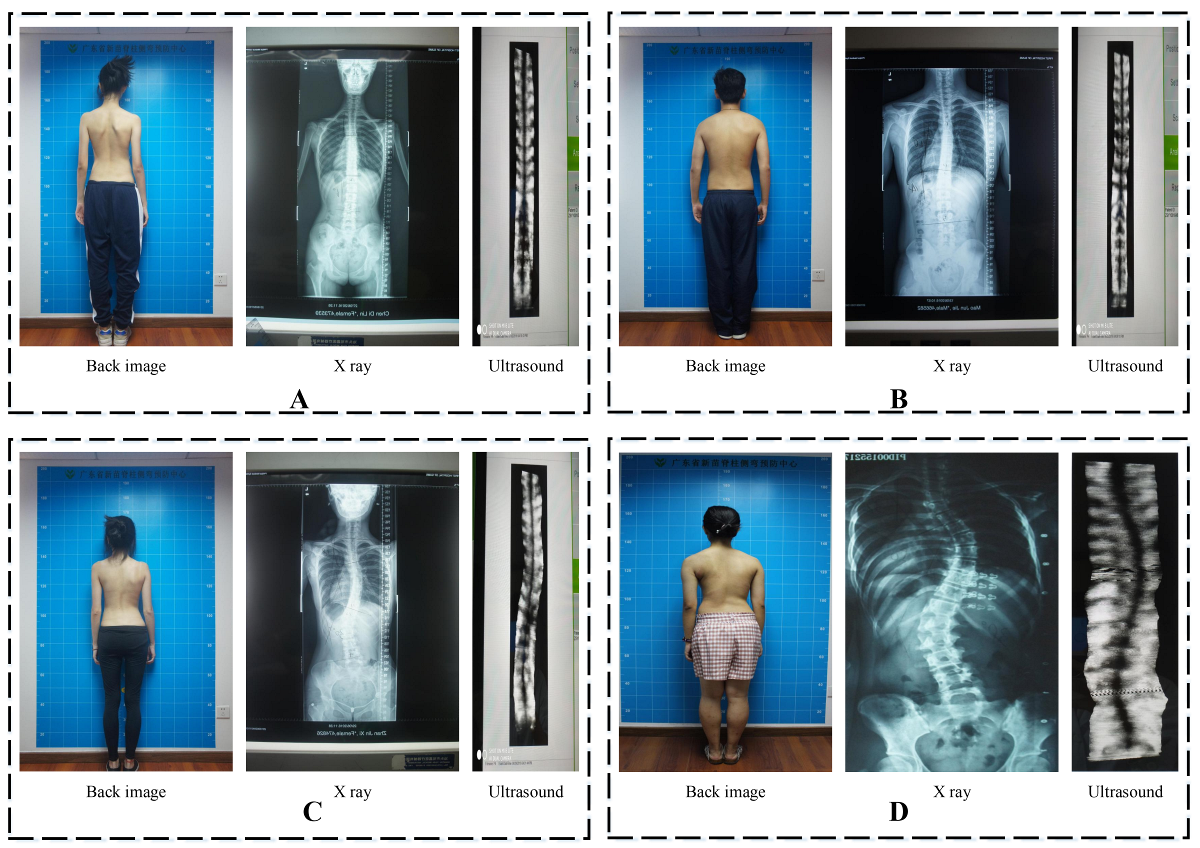


**Supplementary Figure 4. Samples with different curve severities in the datasets. A**, 0 - 9°; **B**, 10 - 19°; **C**, 20 - 44°; **D**, ≥ 45°.

**Supplementary Table 1. Experimental indicators for the two binary classifications and four-class classification with the internal validation dataset.**

| **DLAs** | Algorithm 1 | Algorithm 2 | Algorithm 3 |
| --- | --- | --- | --- |
| **PPV**  **(SD)** | 0.8618  (0.0223) | 0.8912  (0.0172） |  |
| **NPV**  **(SD)** | 0.8585  (0.0328 | 0.8635  (0.0120) |  |
| **Specificity**  **(SD)** | 0.8349  (0.0299) | 0.8961  (0.0172) |  |
| **Sensitivity**  **(SD)** | 0.8751  (0.0322) | 0.8571  (0.0120) |  |
| **Accuracy**  **(SD)** | 0.8561  (0.0262) | 0.8761  (0.0143) | 0.7995  (0.0108) |
| **AUC**  **(SD)** | 0.9455  (0.0151) | 0.9514  (0.0096) |  |

**Supplementary Table 2. Experimental indicators for the two binary classifications and four-class classification with the external validation dataset.** This is the source data used to draw Figure 4(F).

|  | **DLAs** | **Screener 1** | **Screener 2** | **Screener 3** | **Screener 4** | **The Mean (SD) of the Screeners** | |
| --- | --- | --- | --- | --- | --- | --- | --- |
| **Task 1** | **Detecting scoliosis** | | | | | | |
| **Accuracy** | 0.75 | 0.815 | 0.58 | 0.7475 | 0.755 | 0.7244 (0.1009) | |
| **Sensitivity** | 0.8067 | 0.8833 | 0.627 | 0.8 | 0.78 | 0.7726 (0.1069) | |
| **Specificity** | 0.58 | 0.61 | 0.44 | 0.59 | 0.65 | 0.5725 (0.0918) | |
| **NPV** | 0.5 | 0.6354 | 0.2821 | 0.4958 | 0.5078 | 0.4803 (0.1464) | |
| **PPV** | 0.8521 | 0.8717 | 0.7737 | 0.8541 | 0.8713 | 0.8427 (0.0467) | |
| **Task 2** | **Detecting scoliosis cases with curves ≥ 20°** | | | | | | |
| **AUC** | 0.929 |  |  |  |  |  |  |
| **Accuracy** | 0.87 | 0.85 | 0.7775 | 0.8125 | 0.8375 | 0.8194 (0.032) | |
| **Sensitivity** | 0.84 | 0.815 | 0.565 | 0.79 | 0.73 | 0.725 (0.1125) | |
| **Specificity** | 0.9 | 0.885 | 0.99 | 0.835 | 0.945 | 0.9138 (0.0679) | |
| **NPV** | 0.8491 | 0.8271 | 0.6947 | 0.799 | 0.8112 | 0.783 (0.06) | |
| **PPV** | 0.8936 | 0.8763 | 0.9826 | 0.8272 | 0.93 | 0.904 (0.0671) | |
| **Task 3** | **Severity grading of scoliosis** | | | | | | |
| **AUC** |  |  |  |  |  |  |  |
| **Accuracy** | 0.555 | 0.5675 | 0.3375 | 0.4975 | 0.4725 | 0.4688 (0.0963) | |
| **Sensitivity** |  |  |  |  |  |  |  |
| **Specificity** |  |  |  |  |  |  |  |
| **NPV** |  |  |  |  |  |  |  |
| **PPV** |  |  |  |  |  |  |  |

**Supplementary Table 3. Experimental indicators for the four additional classifications. This is the source data used to draw Figures 3(B) and 3(D).**

| **Classifications** | **Accuracy** | **Sensitivity** | **Specificity** | **NPV** | **PPV** | **AUC** |
| --- | --- | --- | --- | --- | --- | --- |
| **Four classes of classifications:**  **(0 - 9°, 10 - 24°, 25 - 44°, ≥ 45°)** | 0.7682  (0.0125) | ------- | ------- | ------- | ------- | ------- |
| **Binary classification:**  **(20 - 44°, ≥ 45°)** | 0.9382  (0.0201) | 0.9311  (0.0250) | 0.9452  (0.0239) | 0.9298  (0.0252) | 0.9468  (0.0226) | 0.9791  (0.0086) |
| **Binary classification:**  **(25 - 44°, ≥ 45°** | 0.9304  (0.00330 | 0.9198  (0.0119) | 0.9410  (0.0115) | 0.9209  (0.0099) | 0.9400  (0.0104) | 0.9761  (0.0071) |
| **Binary classification:**  **(0 - 44°, ≥ 45°)** | 0.9757  (0.0049) | 0.9740  (0.0216) | 0.9766  (0.0117) | 0.9789  (0.0172) | 0.9733  (0/0130) | 0.9960  (0.0015) |

**Supplementary Table 4. Numbers of images with different curve severities in the datasets.**

| **Algorithm for training and validation** | **Different curve severities** | **Numbers of images** | |
| --- | --- | --- | --- |
|  |  | **Datasets for training and internal validation** | **Datasets for external validation** |
| Algorithm 1 | 0 - 9° | 745 | 100 |
|  | ≥ 10° | 825 | 300 |
| Algorithm 2 | 0 - 19° | 1483 | 200 |
|  | ≥ 20° | 1470 | 200 |
| Algorithm 3 | 0 - 9° | 745 | 100 |
|  | 10 - 19° | 738 | 100 |
|  | 20 - 44° | 720 | 100 |
|  | ≥ 45° | 750 | 100 |
| Total | ------ | 3240 | 400 |

**Supplementary Table 5. The matrix shown below was used to draw Figure 2(C).**

| 0.77 | 0.17 | 0.06 | 0 |
| --- | --- | --- | --- |
| 0.07 | 0.74 | 0.18 | 0 |
| 0.06 | 0.16 | 0.78 | 0 |
| 0 | 0 | 0.03 | 0.96 |

**Supplementary Table 6. The matrix shown below is used to draw Figure 4(A).**

| 0.31 | 0.48 | 0.2 | 0.01 |
| --- | --- | --- | --- |
| 0.32 | 0.54 | 0.13 | 0.02 |
| 0.04 | 0.23 | 0.56 | 0.17 |
| 0.01 | 0 | 0.18 | 0.81 |

**Supplementary Table 7. The matrix shown below is used to draw Figure 4(B).**

| 0.65 | 0.28 | 0.07 | 0 |
| --- | --- | --- | --- |
| 0.45 | 0.5 | 0.05 | 0 |
| 0.12 | 0.4 | 0.41 | 0.07 |
| 0 | 0.1 | 0.28 | 0.71 |

**Supplementary Table 8. The matrix shown below is used to draw Figure 4(C).**

| 0.98 | 0.02 | 0 | 0 |
| --- | --- | --- | --- |
| 0.98 | 0 | 0.01 | 0 |
| 0.85 | 0.12 | 0.03 | 0 |
| 0 | 0.23 | 0.43 | 0.34 |

**Supplementary Table 9. The matrix shown below is used to draw Figure 4(D).**

| 0.42 | 0.42 | 0.16 | 0 |
| --- | --- | --- | --- |
| 0.36 | 0.62 | 2 | 0 |
| 0.05 | 0.5 | 0.38 | 0.07 |
| 0 | 0 | 0.43 | 0.57 |

**Supplementary Table 10. The matrix shown below is used to draw Figure 4(E).**

| 0.58 | 0.34 | 0.08 | 0 |
| --- | --- | --- | --- |
| 0.45 | 0.51 | 0.04 | 0 |
| 0.25 | 0.52 | 0.23 | 0 |
| 0 | 0.01 | 0.42 | 0.57 |

.

**Supplementary Table 11. The matrix shown below is used for drawing Supplementary Figure 1(A).**

| 0.70 | 0.23 | 0.06 | 0.013 |
| --- | --- | --- | --- |
| 0.09 | 0.76 | 0.14 | 0.01 |
| 0.03 | 0.18 | 0.74 | 0.05 |
| 0 | 0.04 | 0.05 | 0.93 |
