# Supplementary material for: Development and validation of deep learning algorithms for scoliosis screening using back images
Source: Commun Biol. 2019 Oct 25;2:390. doi: 10.1038/s42003-019-0635-8 (PMC6814825; doi:10.1038/s42003-019-0635-8)
Supplement: Supplementary file 2 — Description of Additional Supplementary Files [file 42003_2019_635_MOESM2_ESM.docx]

This document describes the source data underlying Figures 2-4 and Supplementary Figure 1. The first and second rows of ROC excel files represent the x and y coordinates respectively. You can find ROC1-ROC7 sheets in Supplementary Data 1.xls.

**Source data for Figure 2**

The sheet ‘ROC1’ includes the data points for drawing Figure 2(A).

The sheet ‘ROC2’ includes the data points for drawing Figure 2(B).

**Source data for Figure 3**

The sheet ‘ROC3’ includes the data points for drawing Figure 3(A).

The data used to draw Figure 3(B) is shown in Supplementary Table 2.

The sheet ‘ROC4’ includes the data points for drawing Figure 3(C).

The data used to draw Figure 3(D) is shown in Supplementary Table 2.

**Source data for Supplementary Figure 1**

The sheet ‘ROC5’ includes the data points for drawing Supplementary Figure 1(B).

The sheet‘ROC6’ includes the data points for drawing Supplementary Figure 1(C).

The sheet ‘ROC7’ includes the data points for drawing Supplementary Figure 1(D).
