# Supplementary material for: Development and validation of deep learning algorithms for scoliosis screening using back images
Source: Commun Biol. 2019 Oct 25;2:390. doi: 10.1038/s42003-019-0635-8 (PMC6814825; doi:10.1038/s42003-019-0635-8)
Supplement: Supplementary file 4 — Reporting Summary [file 42003_2019_635_MOESM4_ESM.pdf]

nature research

Corresponding author(s): Junlin Yang, Haotian Lin

Last updated by author(s): Sep 5, 2019

Reporting Summary

Nature Research wishes to improve the reproducibility of the work that we publish. This form provides structure for consistency and transparency in reporting. For further information on Nature Research policies, see Authors & Referees and the Editorial Policy Checklist.

Statistics

For all statistical analyses, confirm that the following items are present in the figure legend, table legend, main text, or Methods section.

n/a	Confirmed	
☐	☒	The exact sample size (n) for each experimental group/condition, given as a discrete number and unit of measurement
☐	☒	A statement on whether measurements were taken from distinct samples or whether the same sample was measured repeatedly
☐	☒	The statistical test(s) used AND whether they are one- or two-sided<br>*Only common tests should be described solely by name; describe more complex techniques in the Methods section.*
☒	☐	A description of all covariates tested
☒	☐	A description of any assumptions or corrections, such as tests of normality and adjustment for multiple comparisons
☐	☒	A full description of the statistical parameters including central tendency (e.g. means) or other basic estimates (e.g. regression coefficient) AND variation (e.g. standard deviation) or associated estimates of uncertainty (e.g. confidence intervals)
☐	☒	For null hypothesis testing, the test statistic (e.g. F, t, r) with confidence intervals, effect sizes, degrees of freedom and P value noted<br>*Give P values as exact values whenever suitable.*
☒	☐	For Bayesian analysis, information on the choice of priors and Markov chain Monte Carlo settings
☒	☐	For hierarchical and complex designs, identification of the appropriate level for tests and full reporting of outcomes
☒	☐	Estimates of effect sizes (e.g. Cohen's d, Pearson's r), indicating how they were calculated

Our web collection on statistics for biologists contains articles on many of the points above.

Software and code

Policy information about availability of computer code

Data collection	No software was used.
Data analysis	For 5-fold cross validation and external validation, the sensitivity, specificity, accuracy, and area under the receiver operating characteristic (AUC) curve of the deep learning algorithms were calculated. The number of true-positive (TP), true-negative (TN), false-positive (FP), and false-negative (FN) cases were counted, and the corresponding PPV and negative predictive value (NPV) were calculated. The performances of the deep learning algorithms in the external validation were compared with those of professional screeners using descriptive statistics. All of the statistical tests in our study were 2-sided, and a P-value less than 0.05 was considered significant. All analyses were performed with MATLAB (Version R2016a, MathWorks, http://www.mathworks.com).

For manuscripts utilizing custom algorithms or software that are central to the research but not yet described in published literature, software must be made available to editors/reviewers. We strongly encourage code deposition in a community repository (e.g. GitHub). See the Nature Research guidelines for submitting code & software for further information.

Data

Policy information about availability of data

All manuscripts must include a data availability statement. This statement should provide the following information, where applicable:

- Accession codes, unique identifiers, or web links for publicly available datasets
- A list of figures that have associated raw data
- A description of any restrictions on data availability

The data in this study include the training dataset and the test dataset. Correspondence and requests for data materials should be addressed to JLY (yjunlin@126.com).

Field-specific reporting

Please select the one below that is the best fit for your research. If you are not sure, read the appropriate sections before making your selection.

☒ Life sciences ☐ Behavioural & social sciences ☐ Ecological, evolutionary & environmental sciences

For a reference copy of the document with all sections, see nature.com/documents/nr-reporting-summary-flat.pdf

Life sciences study design

All studies must disclose on these points even when the disclosure is negative.

Sample size	The training dataset included 3,240 patients with labeled back images and whole-spine standing posterior-anterior X-ray images or ultrasound images from the databases of three institutions, 2,495 of which were obtained from subjects with scoliosis and 745 of which were obtained from normal controls. The datasets used for external validation consisted of the data from 400 individuals, including 100 normal cases and 300 scoliosis cases.
Data exclusions	No data were excluded.
Replication	To detect cases with a curve ≥ 10°, algorithm 1 exhibited an average AUC of 0.946 (95% CI, 0.916 - 0.975), a sensitivity of 87.5% (95% CI, 81.2% - 93.8%), a specificity of 83.5% (95% CI, 77.6% - 89.4%), and a PPV of 86.2% (95% CI, 81.6% - 90.8%). For algorithm 2, the AUC, sensitivity, and specificity to detect cases with a curve ≥20° were 0.951 (95% CI, 0.933 - 0.970), 85.7% (95% CI, 83.4% - 88.1%), and 89.6% (95% CI, 86.2% - 93.0%), respectively, with a PPV of 89.1% (95% CI, 85.8% - 92.5%). The average accuracy of algorithm 3 was 80.0% (95% CI, 77.8% - 82.1%) for differentiating among the four groups. The internal validation results are shown in Figure 2 and Supplementary Table 2.
Randomization	The experiments were not randomized.
Blinding	Blinding was not used for the experiments.

Reporting for specific materials, systems and methods

We require information from authors about some types of materials, experimental systems and methods used in many studies. Here, indicate whether each material, system or method listed is relevant to your study. If you are not sure if a list item applies to your research, read the appropriate section before selecting a response.

Materials & experimental systems

n/a	Involved in the study	
☒	☐	Antibodies
☒	☐	Eukaryotic cell lines
☒	☐	Palaeontology
☒	☐	Animals and other organisms
☐	☒	Human research participants
☐	☒	Clinical data

Methods

n/a	Involved in the study	
☒	☐	ChIP-seq
☒	☐	Flow cytometry
☒	☐	MRI-based neuroimaging

Human research participants

Policy information about studies involving human research participants

Population characteristics	The training dataset included 3,240 patients with labeled back images and whole-spine standing posterior-anterior X-ray images or ultrasound images from the databases of three institutions, 2,495 of which were obtained from subjects with scoliosis and 745 of which were obtained from normal controls. The datasets used for external validation consisted of the data from 400 individuals, including 100 normal cases and 300 scoliosis cases.
Recruitment	The back images for algorithm training and validation were collected from patients screened or treated for scoliosis at three institutions (Xinhua Hospital, Xinmiao Spine Clinic, and the 1st Affiliated Hospital of Sun Yat-sen University).
Ethics oversight	Our study was approved by the institutional review board (IRB: XHEC-KJB-2018-024) and was conducted in accordance with the Declaration of Helsinki.

Note that full information on the approval of the study protocol must also be provided in the manuscript.

Clinical data

Policy information about clinical studies

All manuscripts should comply with the ICMJE guidelines for publication of clinical research and a completed CONSORT checklist must be included with all submissions.

Clinical trial registration	NCT03773458
Study protocol	The test between the DLAs and the orthopedists was registered with ClinicalTrials.gov (identifier: NCT03773458).
Data collection	The datasets used for external validation consisted of the data from 400 individuals, including 100 normal cases and 300 scoliosis cases. The requirement for informed consent was waived because of the retrospective nature of the fully anonymized images.
Outcomes	The DLAs exhibited higher accuracy than the human specialists in detecting scoliosis (algorithm 1, 75.0%; human, 72.4%) and identifying cases with a curve ≥ 20°, which require a brace or surgical treatment (algorithm 2, 87%; human, 81.9%). The accuracy of algorithm 3 was 55.5% for differentiating among the four groups, which was comparable to the highest accuracy (56.8%) of the four human experts and superior to the mean level (46.9%).
